# Cardiac contractility modulation ameliorates myocardial metabolic remodeling in a rabbit model of chronic heart failure through activation of AMPK and PPAR-α pathway

**DOI:** 10.1515/med-2022-0415

**Published:** 2022-02-22

**Authors:** Feifei Zhang, Litian Liu, Yuetao Xie, Jiaqi Wang, Xuefeng Chen, Shihang Zheng, Yingxiao Li, Yi Dang

**Affiliations:** Department of Cardiology Center, Hebei General Hospital, Xipingxilu 348, Xinhua, Shijiazhuang, 050051, Hebei Province, China

**Keywords:** cardiac contractility modulation, heart failure, metabolic remodeling

## Abstract

Metabolic remodeling contributes to the pathological process of heart failure (HF). We explored the effects of cardiac contractility modulation (CCM) on myocardial metabolic remodeling in the rabbit model with HF. The HF in rabbit model was established by pressure uploading and then CCM was applied. We evaluated the cardiac structure and function by echocardiography, serum BNP level, and hematoxylin and eosin and Masson’s trichrome staining. We detected the accumulation of glycogen and lipid droplets in myocardial tissues by periodic acid-Schiff and Oil Red O staining. Then, we measured the contents of glucose, free fatty acid (FFA), lactic acid, pyruvate, and adenosine triphosphate (ATP) levels in myocardial tissues by corresponding kits and the expression levels of key factors related to myocardial substrate uptake and utilization by western blotting were analyzed. CCM significantly restored the cardiac structure and function in the rabbit model with HF. CCM therapy further decreased the accumulation of glycogen and lipid droplets. Furthermore, CCM reduced the contents of FFA, glucose, and lactic acid, and increased pyruvate and ATP levels in HF tissues. The protein expression levels related to myocardial substrate uptake and utilization were markedly improved with CCM treatment by further activating adenosine monophosphate-activated protein kinase and peroxisome proliferator-activated receptor-α signaling pathways.

## 1 Introduction

Heart failure (HF) is one of the leading causes of death worldwide and continues to increase in prevalence throughout the world. The drug therapies including, beta-blockers, renin-angiotensin system inhibitor, mineralocorticoid receptor antagonists, ivabradine, sodium-glucose cotransporter 2 inhibitors, vericiguat, and device therapies, including cardiac resynchronization therapy, left bundle branch area pacing, and ventricular assist devices for HF, have made impressive progress in the last decade [1]. Despite these therapeutic and technological advancements in managing the morbidity and mortality in HF patients, a considerable subset of patients are not eligible for either of these pre-established therapies, which often make the prognostic outcomes challenging for these patients [2]. Therefore, there is an urgent requirement to explore new therapeutic targets and strategies for managing patients with HF.

Emerging studies have suggested that altered energy metabolism, energy deficiency due to substrate utilization and metabolism derangements, and oxidative stress are the pathological hallmarks of cardiovascular diseases, including HF [3,4]. Under normal physiological conditions, the heart prefers to derive required energy by the fatty acid oxidation pathway as the predominant source of adenosine triphosphate (ATP) production, while carbohydrates can provide only a part of the essential amount of ATP [5]. Because of the loss of metabolic flexibility, the failing heart switches its energy metabolism and substrate utilization preferences from high-energy fatty acids to other alternative substrates, resulting in a significant lack of ATP production, thus prompting the pathophysiological processes of HF [6,7]. These changes in fuel selection are driven by a reprogramming of the metabolic pathway-related gene expression pattern in the failing heart [3]. Previously reported experimental and clinical findings suggest that targeting the substrate utilization mechanism may be a promising therapeutic avenue in managing HF [8].

Cardiac contractility modulation (CCM) is an electrical modality for the treatment of HF, that delivers relatively high voltage and long duration biphasic electrical impulses to the myocardium during the absolute refractory period; therefore, it neither influences the heart rhythm nor the normal propagation of the cardiac action potential across the valves [9,10]. CCM has been shown to increase the ventricular contractility with resultant improvement in the functional capacity of the heart, without the need for an additional oxygen request [11]. The underlying mechanism(s) of action is not completely understood yet; however, it appears to be multifactorial at both cellular and molecular levels, with improved calcium handling and restoration of normal gene expression pattern [12]. We have previously demonstrated that CCM can exert its protective effects against myocardial fibrosis by potentially inhibiting transforming growth factor-β (TGF-β)/Smad3 signaling pathway [13]. Studies have shown that therapy with antifibrotic agents may have metabolic implications in HF [14]. TGF-β signaling plays a significant role in cellular metabolic activities and energy homeostasis by interacting with peroxisome proliferator-activated receptor-α (PPAR-α) and regulating the expression of key enzymes involved in the substrate utilization processes [15,16]. However, no direct evidence suggests the implication of CCM on the modulation of high-energy phosphate production in the skeletal and cardiac muscles in HF. Thus, we hypothesized that with the improvement of cardiac contractility force and the attenuation of fibrosis, CCM could ameliorate cardiac metabolism dysfunction in HF and facilitate the supply of sufficient energy. In this study, the critical factors and pathways related to energy substrate metabolism were measured in the HF rabbit model to elaborate on the protective effects of CCM on HF.

## 2 Methods

### 2.1 Experimental animals and treatment

Six-month-old healthy New Zealand white rabbits weighing 2.5–3.5 kg were obtained from the Experimental Animal Center of Hebei Medical University (Hebei, China). Hebei General Hospital Ethics Committee approved the research protocol. All procedures involving animals were in full compliance with the Guide for the Care and Use of Laboratory Animals published by the US National Institutes of Health (NIH Publication No. 85-23, revised 1996).

In this study, HF was induced in the rabbit model by applying pressure overload through a transverse aortic constriction (TAC) [17]. 3% Sodium pentobarbital (1 mL/kg) was used to anesthetize rabbits by injecting them in the ear vein. The thoracic cavity was opened from the left side of the sternum second intercostal space. Then, the ascending aorta was dissected to make an additional constriction to 60% of the original circumference of the aorta (Figure S1a). Characteristic symptoms of HF, including loss of appetite, reduction in physical activities, and breathing acceleration, were manifested 12 weeks post-surgery. Left ventricular ejection fraction (LVEF) of ≤40% by echocardiography examination indicated the successful establishment of the HF model.

**Figure 1:**
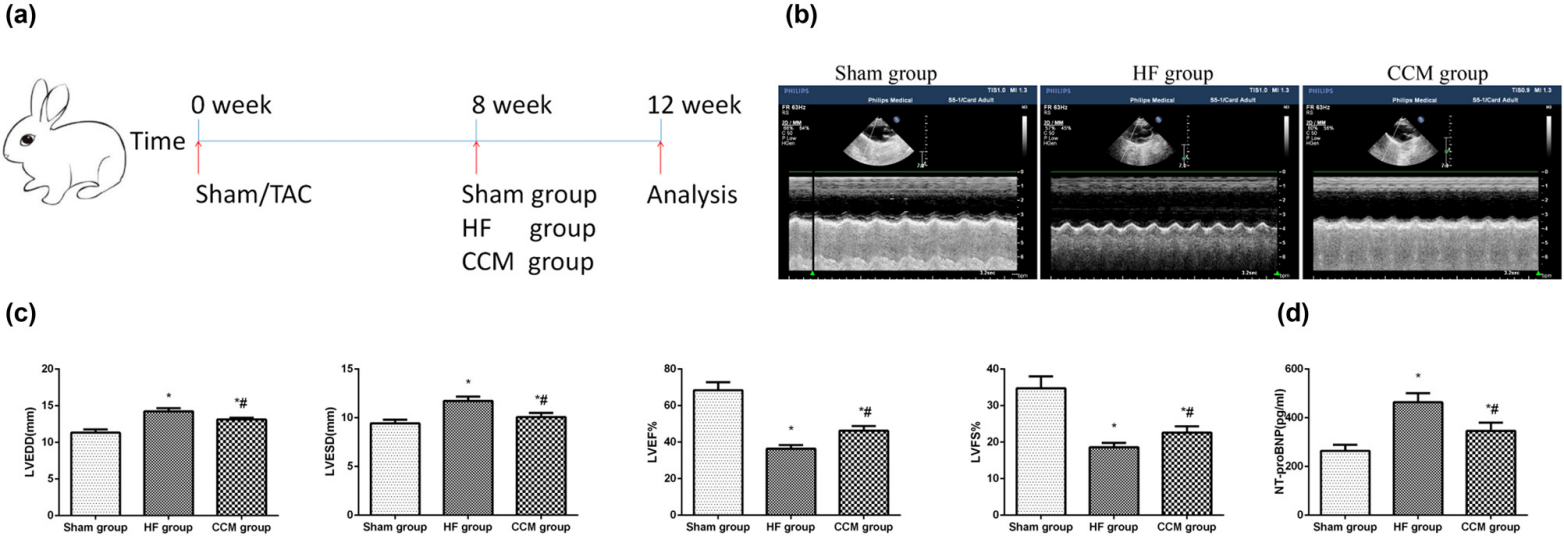
CCM treatment improves cardiac function in a HF rabbit model. (a) A time frame of the 16-week experiment. (b) Representative echocardiograms in different groups. (c) Echocardiography analyses of LVESD, LVEDD, LVEF, and LVFS. (d) Detection of BNP levels by enzyme-linked immunosorbent assay (ELISA). Data are expressed as mean values ± SD (*P < 0.05 compared with the sham group; ^#^ *P* < 0.05 compared with HF group).

All animals were randomly divided into sham (*n* = 10) or TAC group (*n* = 20) at first. Rabbits in the sham group received only thoracotomy, while animals in the TAC group received thoracotomy and TAC. Then, a pediatric temporary pacing lead (6491 Medtronic, Inc, USA) was stitched to the left ventricular anterior wall to deliver CCM signals in the next step. The 18 surviving rabbits that underwent TAC and met the criteria for HF induction were randomly allocated into HF (*n* = 9) and CCM (*n* = 9) groups. Rabbits in the CCM group received the treatment for 4 weeks. The CCM signals were applied to the absolute refractory period of the heart by EPS320 Cardiac Stimulator (BARD Micro Pace, Inc, USA). The signals consisted of biphasic square wave pulses with phase duration = 2 ms, stimulus amplitude = 7 V, and 30 ms delay after R-wave sensing (Figure S2). The CCM signals were applied for 6 h per day for 4 weeks. At the end of the experiment, rabbits were sacrificed by exsanguination under anesthesia, and blood and heart samples were prepared for further investigation (the experimental timeline, Figure 1a).

### 2.2 Echocardiography procedure

Transthoracic echocardiography was used to detect and record cardiac structure and function by IU 22 Ultrasound instrument with a 10 MHz transducer (Philips Healthcare, Best, The Netherlands) under the anesthetic condition with 3% sodium pentobarbital. Cardiac cycles were recorded by the M-mode ultrasound from the long-axis parasternal view. The following indicators were measured: left ventricular end-systolic diameter (LVESD), left ventricular end-diastolic diameter (LVEDD), LVEF, and left ventricular fractional shortening (LVFS).

### 2.3 NT-proBNP measurement

Collected blood samples were centrifuged at 1,500 rpm for 10 min. The serum NT-proBNP levels were then measured by an enzyme-linked immunosorbent assay (USCN Science, Inc, Wuhan, China).

### 2.4 Histopathological analysis

4% Paraformaldehyde (PFA) was used to fix rabbit myocardial tissues, followed by paraffin embedding and tissue dehydration. Then, the tissue sample was cut into 5 mm thick sections. Cardiomyocyte architecture and interstitial fibrosis were assessed by hematoxylin and eosin (H and E) and Masson’s trichrome staining. The sum of the soft connective tissue areas was divided by the sum of the area of all types of connective tissues, and muscle area was calculated as collagen volume fraction (CVF). Accumulation of glycogen and lipid droplets in the cardiac tissue was detected by periodic acid-Schiff (PAS) staining, where glycogen was shown in red granules, and lipid droplets were visible in red color in Oil Red O staining (Nanjing Jiancheng, Inc, Nanjing, China). Images were visualized under an optical microscope at 400× magnification.

### 2.5 Biochemical measurements

Quantitative detection of glucose, free fatty acid (FFA), lactic acid, and pyruvate in the myocardium tissue was carried by colorimetric method (Nanjing Jiancheng, Inc, Nanjing, China). The levels of ATP and lactate in the myocardium were measured by ELISA (USCN Science, Inc, Wuhan, China). All procedures were performed following the manufacturer’s instructions.

### 2.6 Western blot analysis

Cardiac tissues of the left ventricle free wall were quickly frozen in liquid nitrogen and subsequently stored in a freezer at −80°C for further investigation. Western blotting was performed to detect the expression level of key factors related to myocardial substrate uptake and utilization. Proteins were extracted from cardiac tissues, separated with 10% SDS-PAGE electrophoresis and transferred to polyvinylidene difluoride membrane. After blocking with 5% fat-free milk for 2 h, the membrane was incubated with primary antibody overnight at 4°C. Primary antibodies for glucose transporter 4 (GLUT4) (1:1,000, Invitrogen, MA1-83191), hexokinase2 (HK2) (1:1,000, Bioss, bs-9455R), phosphofructokinase6 (PFK6) (Novus, NB2-44228), pyruvate dehydrogenase (PDH) (1:1,000, Sigma, AVA48136), pyruvate dehydrogenase kinase 4 (PDK4) (1:1,000, Novus, NBP1-54723), lactate dehydrogenase (LDH) (1:1,000, Novus, NB600-861), cluster of differentiation 36 (CD36) (1:1,000, Novus, NB400-144), carnitine palmitoyltransferase-1 (CPT-1) (1:1,000, Novus, NBP1-59576), acyl-coA dehydrogenase medium chain (ACADM) (1:1,000, Bioss, BS-4047R), acetyl-coenzyme-A-carboxylase (ACC) (1:1,000, Abcam, ab72046), adenosine monophosphate-activated protein kinase (AMPK) (1:1,000, Novus, BNP1-56322), PPAR-α (1:1,000, Novus, NB300-537), and peroxisome proliferator-activated receptor coactivator 1α (PGC-1α) (1:1,000, Novus, NB300-537) were used to detect specific proteins, and β-actin (1:1,500, Bioss, bs-0061R) was used as the loading control. Finally, the immunoreactive bands were visualized with an enhanced chemiluminescence kit (ECL Millipore Corp., Bedford, MA, USA) and quantified using Image-ProPlus 5.1.

### 2.7 Statistical analysis

Data were presented as mean value ± SD. A one-way analysis of variance (ANOVA) was conducted for comparisons of multiple groups, followed by pairwise comparisons were performed using the Student–Neuman–Keuls test. Statistical analysis was conducted using IBM SPSS 22 (Lead Technologies, Chicago, USA). Figures were prepared in GraphPad Prism 8.0 (GraphPad Software, Inc., La Jolla, CA, USA). A value of *P* < 0.05 was considered statistically significant.

## 3 Results

### 3.1 Effect of CCM on cardiac function and structure in HF animals

**Figure 2:**
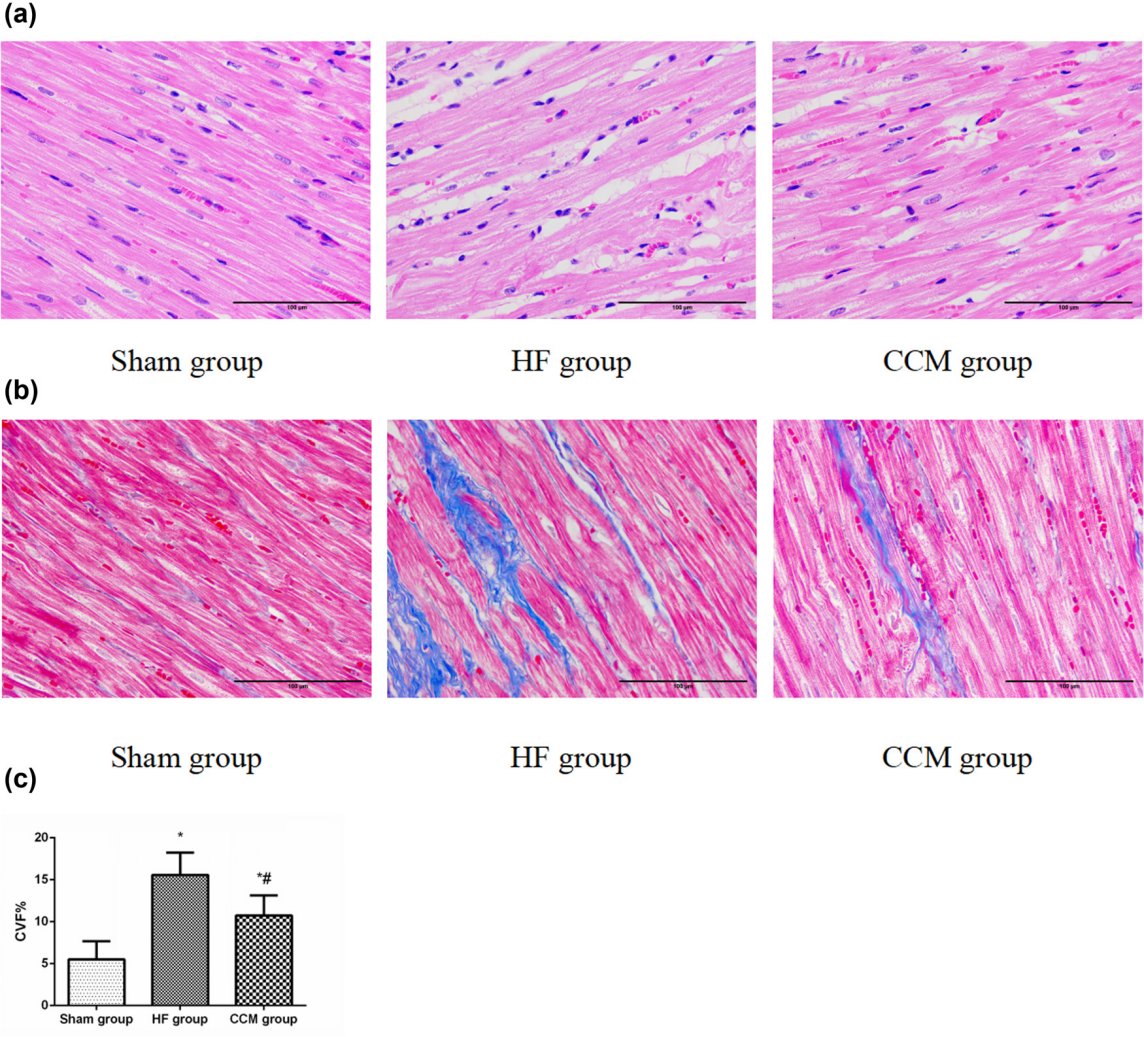
CCM treatment ameliorates pathological changes in cardiac tissues. (a) H and E staining of cardiac tissues (400× magnification). (b) Masson’s trichrome staining of cardiac tissues (400× magnification). (c) The CVF of myocardial tissues was quantitated. Data are expressed as mean values ± SD (*P < 0.05 compared with the sham group; ^#^ *P* < 0.05 compared with HF group).

Here we verified that the TAC rabbits had remarkable impairment of the cardiac function at 12 weeks, manifested as a significant decrease in LVEF and LVFS values, as well as obvious enlargement of the LVEDD and LVESD. In addition, serum BNP levels were significantly elevated after inducing HF (Figure S1b). Treatment with CCM restored the LVESD, LVEDD, and increased the EF and FS significantly compared to the untreated HF group. It also improved the serum N-terminal pro-BNP (NT-proBNP) level in HF animals (Figure 1b–d). Consistently, the morphological assessments by H and E and Masson’s staining revealed that CCM alleviated myocardial necrosis, infiltration of inflammatory cells and myocardial interstitial fibrosis, and restored the physiological arrangement of myocardial cells (Figure 2a–c). Together, these findings suggest that CCM may potentially improve cardiac function and structure in patients with HF.

### 3.2 Effect of CCM on utilization of energy substrate and ATP production in HF animals

**Figure 3:**
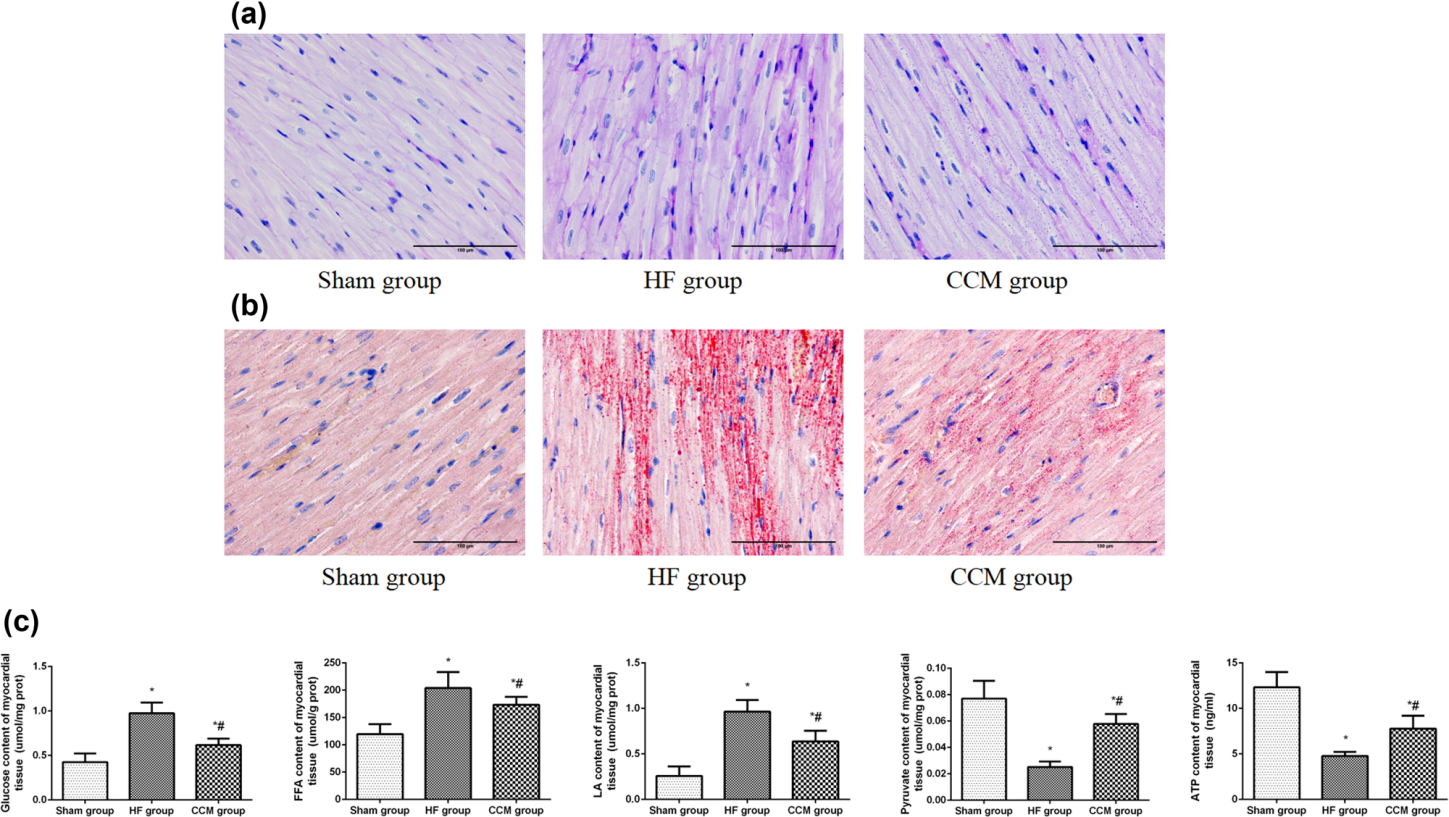
CCM treatment improves the utilization of energy substrate and increased the production of ATP. (a) PAS staining of cardiac tissues (400× magnification). (b) Oil Red O staining of cardiac tissues (400× magnification). (c) Detection of myocardium contents of ATP, glucose, FFA, lactic acid, and pyruvate. Data are expressed as mean values ± SD (*P < 0.05 compared with the sham group; ^#^ *P* < 0.05 compared with HF group).

The accumulation of glycogen and lipid droplets were assessed by PAS and Oil red O staining, respectively, where PAS staining showed a large number of red granules in the HF group’s myocardium, and this accumulation was partially attenuated by CCM treatment. Oil Red O staining revealed that the HF group’s tissue had large numbers of lipid droplets stained with red color, while CCM treatment significantly decreased the lipid droplet count (Figure 3a and b). Myocardium tissues from the HF group showed an increased accumulation of FFA, glucose, and lactic acid. However, there was a decrease in levels of ATP and pyruvate compared to the sham group. In contrast, CCM treatment remarkably prevented the accumulation of FFA and glucose, reduced the production of lactic acid, and improved the condition of ATP insufficiency (Figure 3c). These data showed that the alteration of energy metabolism occurred in HF, in which the rate of glycolysis was increased, and fatty acid oxidation rate was decreased, resulting in the accumulation of glycolipids and lactic acid in the muscle and serum. However, CCM could improve the utilization of energy substrate and increase the production of ATP.

### 3.3 Effect of CCM in regulating the key glucose metabolism factors in HF animals

**Figure 4:**
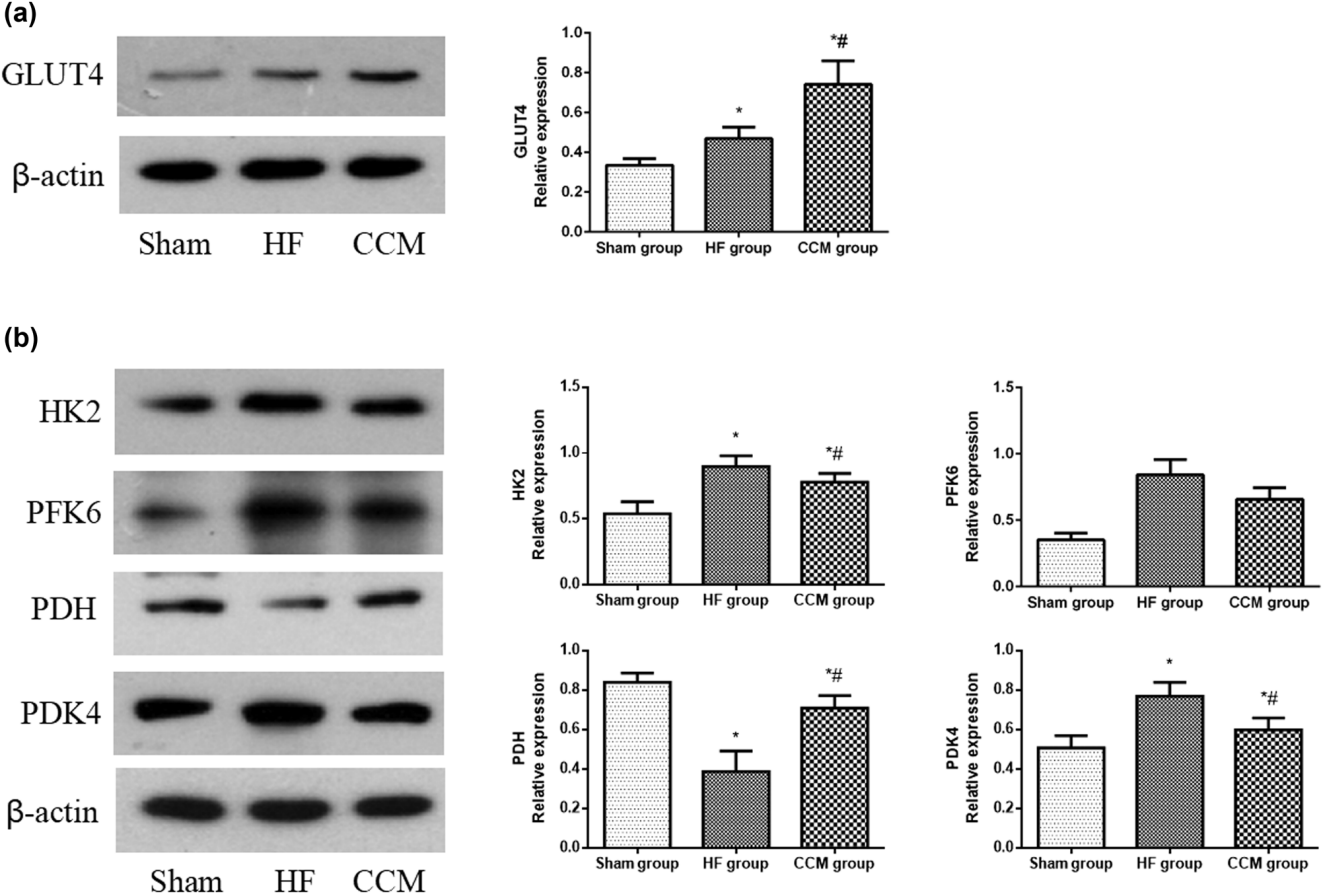
CCM treatment regulates the key glucose metabolism factors in rabbits with HF. (a) Western blot analysis of the expression of GLUT4. Densitometric analysis is shown in the bar graph. (b) Western blot analysis of the expressions of HK2, PFK6, PDH, and PDK4. Densitometric analysis is shown in the bar graph. Data are expressed as mean values ± SD (*P < 0.05 compared with the sham group; ^#^ *P* < 0.05 compared with HF group).

To investigate how CCM regulates glucose metabolism, we measured the expression of key factors in the glucose metabolism pathway, including GLUT4, HK2, PFK6, PDH, and PDK4, by western blotting in the control and experimental groups. GLUT4 transporters are responsible for the bulk of basal glucose uptake in cardiomyocytes. HK2 and PFK6 are rate-limiting enzymes in governing glycolysis. PDH, the rate-limiting enzyme for glucose oxidation, can be inactivated by PDK4. The upregulated expressions of GLUT4, HK2, PFK6, and PDK4 and downregulated expression of PDH in the HF group compared to the sham group suggest a switch toward increased glycolysis rather than enhanced glucose oxidation. In contrast, CCM treatment significantly reduced the expression of PDK4 and LDH and increased the expression of PDH, indicating that CCM inhibited uncoupling of glycolysis from glucose oxidation (Figure 4a and b).

### 3.4 Effect of CCM in regulating the key fatty acid metabolism factors in HF animals

**Figure 5:**
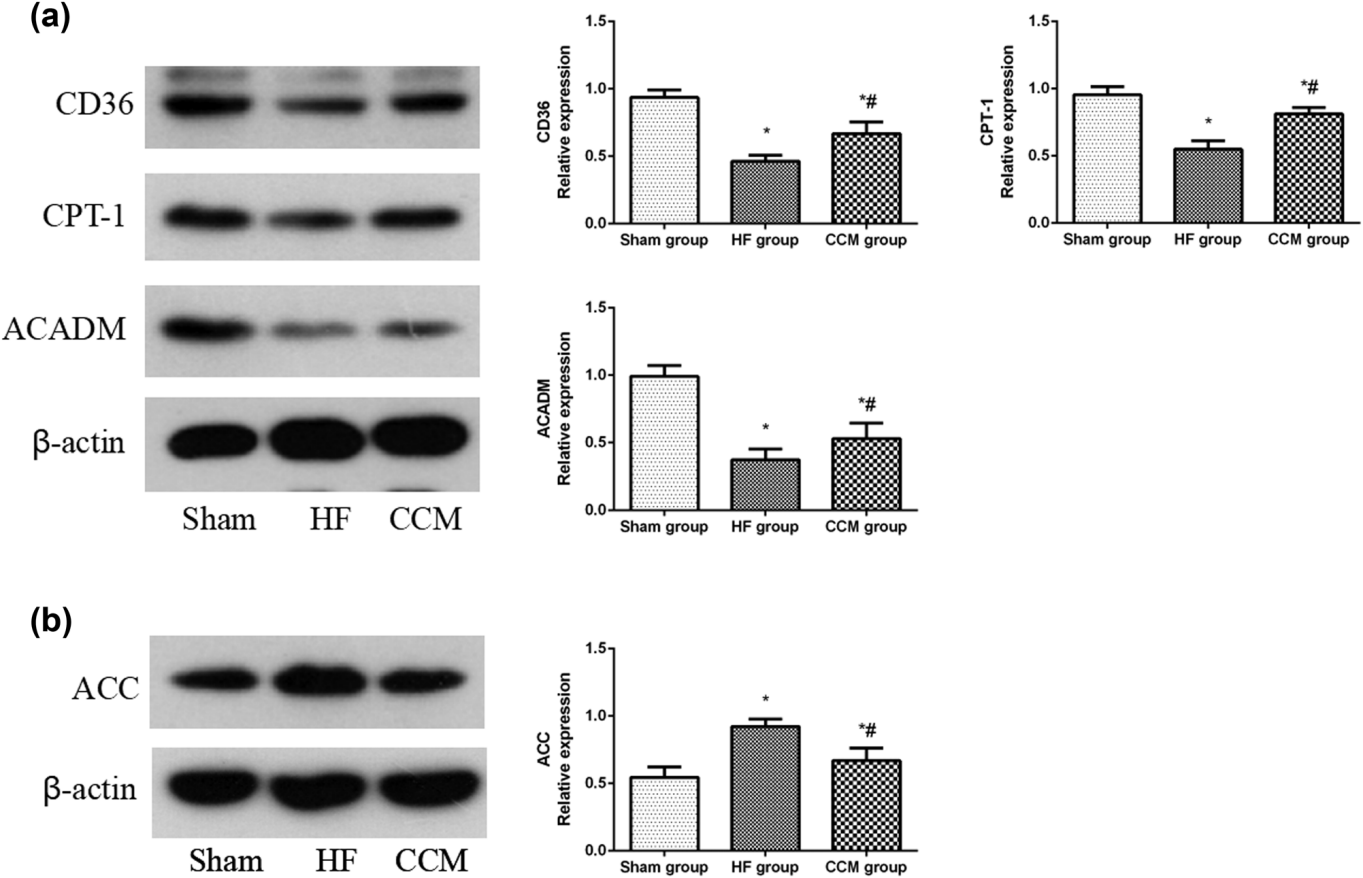
CCM treatment regulates the key fatty acid metabolism factors in rabbits with HF. (a) Western blot analysis of the expressions of CD36, CPT-1, and ADAM. Densitometric analysis is shown in the bar graph. (b) Western blot analysis of the expression of ACC. Densitometric analysis is shown in the bar graph. Data are expressed as mean values ± SD (*P < 0.05 compared with the sham group; ^#^ *P* < 0.05 compared with HF group).

Since we found evidence for the dysregulated lipid metabolism in HF animals, we further examined the protein expression levels of key FFA metabolism-related factors, including CD36, CPT-1, ACADM, and ACC. The protein levels of cardiac CD36, CPT-1, and ACADM were significantly downregulated, and that of ACC was upregulated in the HF group compared to the sham group, suggesting that the ability of FFA intake, transportation, and β-oxidation were severely impaired. However, treatment with CCM remarkably increased the expression of CD36, CPT-1, and ACADM, while reduced the expression of ACC. Together, these results imply that CCM could increase the utilization of FFA (Figure 5a and b).

### 3.5 Effect of CCM on AMPK and PPAR-α pathway in rabbits with HF

**Figure 6:**
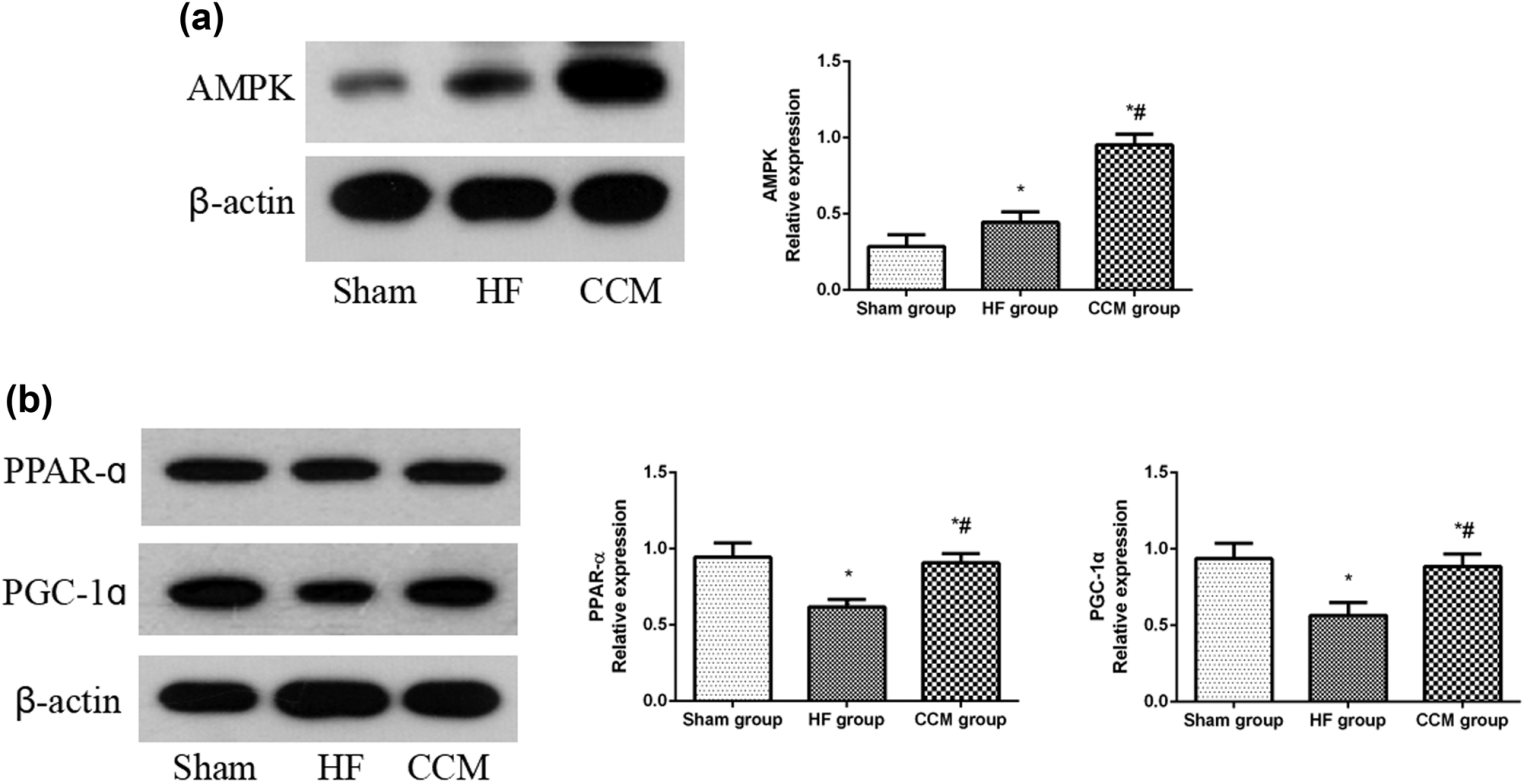
CCM treatment upregulates AMPK and PPAR-α pathways. (a) Western blot analysis of the expression of AMPK. Densitometric analysis is shown in the bar graph. (b) Western blot analysis of the expressions of PPAR-α and PGC-1α. Densitometric analysis is shown in the bar graph. Data are expressed as mean values ± SD (*P < 0.05 compared with the sham group; ^#^ *P* < 0.05 compared with HF group).

AMPK regulates cardiac energy homeostasis and is considered a sensor of metabolic stress. PPAR-α, a downstream effector of AMPK, could affect the expression of key factors related to energy substrate metabolism. Compared to the sham group, the expression of AMPK was upregulated, while the expressions of PPAR-α and PGC-1α were downregulated in the HF group animals. However, CCM intervention further increased AMPK, PPAR-α, and PGC-1α expressions (Figure 6a and b). These results indicate that CCM could activate the AMPK and PPAR-α pathways and promote the expression of key factors related to myocardial substrate uptake and utilization.

## 4 Discussion

Here we investigated the effects of CCM therapy on myocardium energy metabolism in a HF rabbit model as well as its underlying mechanisms. The major findings were as follows: (1) CCM therapy improved cardiac function and structure in a rabbit model with HF; (2) CCM therapy increased the production of ATP by inhibiting the uncoupling of glycolysis from glucose oxidation and increased utilization of FFA; and (3) the underlying mechanism might involve regulation of AMPK and PPAR-α pathways.

CCM could improve myocardium contractility, exercise tolerance, and HF symptoms, which have been developed as a treatment for HF. The mechanism of CCM is not yet completely understood and appears to be multifactorial, involving the regulation of Ca^2+^ signaling mainly by sarcoplasmic reticulum Ca-ATPase (SERCA) and other pathways involved in myocyte and interstitial fibrosis [18,19]. Consistent with previous findings [13,20], CCM therapy attenuated myocardial fibrosis and improved cardiac function. Enough ATP is an indispensable fuel to support the activity of SERCA2A to regulate Ca^2+^ signaling in the sarcoplasmic reticulum and sustain the strong contractile force. However, myocardial metabolic deterioration is the intrinsic hallmark of HF and results in a progressive loss of ATP. Basic research and clinical studies have indicated that CCM may improve Ca^2+^ handling in cardiomyocytes and is not associated with an increase in myocardial oxygen consumption [21,22]. In addition, robust data revealed that therapeutically targeting myocardial fibrosis could reverse HF-associated metabolic alterations [14,23]. Hence, we hypothesized that CCM could affect cardiac energy metabolism in HF and improve heart energy supply.

HF is accompanied by derangements of substrate utilization and intermediate metabolism. Under normal conditions, fatty acid oxidation (FAO) is the primary source of energy production since glycolysis makes only a small fraction of the total ATP pool. However, both glucose intake and glycolysis rates are significantly increased to compensate for the decrease in the FAO in HF [24,25]. Notably, we found consistent results in this study, as increased glucose uptake by the myocardial cells due to the high expression of GLUT4 in HF. Lactic acid and pyruvate, the main intermediates of glucose metabolism in the cytoplasm, alter their normal physiological directions of energy metabolism due to the upregulation of HK2, PFK6, LDH, and PDK4, and downregulation of PDH in HF. While glucose uptake into cardiomyocytes was increased, energy substrate ingestion into the mitochondrial energy metabolism system was subsequently decreased. This might contribute to a notable reduction in glucose oxidation and an increase in glycolysis. The compromised FAO could be related to the abnormal expression of CD36, CPT-1, ACADM, and ACC, leading to the accumulation of fatty acid in the myocardium. However, CCM intervention alleviated the disturbance of glucose and lipid metabolisms by regulating the key factors in substrate metabolism, which contributed to a decrease in FFA, glucose, and lactic acid levels as well as the increase in ATP content in the myocardium.

AMPK functions as an important regulator of metabolic homeostasis. It has been shown that HF results in an increase in AMPK activity. Activation of AMPK could increase ATP synthesis through stimulation of glucose uptake, FAO, and glycolysis, thereby inhibiting energy-consuming anabolic pathways [26,27]. However, during the course of disease progression, compensatory responses of AMPK activation may not be able to satisfy various physiological demands for energy, which can induce pathological changes [28]. Recent investigations suggest that under the circumstance of upregulated AMPK in HF, further AMPK activation could ameliorate myocardial metabolic remodeling [29]. Fortunately, CCM therapy indeed relieved these abnormal metabolic changes in HF by activating AMPK in the current study. Moreover, AMPK-mediated transportation of GLUT4 from the cytosol to the membrane might increase glucose uptake and promote the upregulated PFK6-mediated glycolysis. AMPK can also promote fatty acid uptake and oxidation by increasing the activity of CPT-1 and inhibiting the activity of ACC. These results were also further validated in this study.

PPAR-α plays a critical role in synergistic regulation of cardiac metabolism through transcriptional control. Together with its coactivator PGC-1α, PPAR-α binds to the PPAR response element to activate the expression of genes involved in cardiac fatty acid utilization, including CD36, CPT-1, and ACADM [30,31]. Cardiac levels of PPAR-α and PGC-1α have been shown to decrease in association with the decreased expression of target genes in HF [32]. It has been proved that PPAR-α activation during the pressure overloaded HF induction can improve cardiac function and energy. In addition, PPAR-α also participates in the signaling cascades driven by AMPK [28,33,34]. In accordance with previous studies, PPAR-α, PGC-1α, CD36, CPT-1, and ACADM levels were decreased in the HF group, compared to that in the sham control group. However, the pathological alterations were reversed by CCM treatment.

The study showed that metabolic remodeling was accompanied by myocardial structure and function damage in HF rabbits. CCM therapy could promote the expression of key factors related to metabolic substrate uptake and utilization via the activity of AMPK and PPAR-α pathways. This regulation ameliorated the reduction in ATP production and a deterioration in cardiac structure and function.
